# From Anatase TiO_2_ Nano-Cuboids to Nano-Bipyramids: Influence of Particle Shape on the TiO_2_ Photocatalytic Degradation of Emerging Contaminants in Contrasted Water Matrices

**DOI:** 10.3390/molecules30020424

**Published:** 2025-01-20

**Authors:** Humaira Asghar, Daphne Hermosilla, Francesco Pellegrino, Virginia Muelas-Ramos, Christian de los Ríos, Antonio Gascó, Valter Maurino, Muhammad Ahsan Iqbal

**Affiliations:** 1 Department of Chemistry and UNITO-ITT Joint Lab, University of Torino, Via Giuria 7, 10125 Torino, Italy; humaira.asghar@unito.it (H.A.); francesco.pellegrino@unito.it (F.P.); 2 G-Aqua Research Group, Departamento de Ingeniería y Gestión Forestal y Ambiental, Escuela Técnica Superior de Ingeniería de Montes, Forestal y del Medio Natural, Universidad Politécnica de Madrid, C/José Antonio Novais 10, 28040 Madrid, Spain; daphne.hermosilla@upm.es (D.H.); virginia.muelas@upm.es (V.M.-R.); christian.delosrios.quinones@alumnos.upm.es (C.d.l.R.); antonio.gasco@upm.es (A.G.); 3 Departamento de Ingeniería Química y de Materiales, Facultad de Ciencias Químicas, Universidad Complutense de Madrid, 28040 Madrid, Spain; miqbal@ucm.es

**Keywords:** shape-controlled TiO_2_, nano-cuboids, photocatalysis, dyes, diclofenac, methomyl, stormwater

## Abstract

Water pollution, resulting from industrial effluents, agricultural runoff, and pharmaceutical residues, poses serious threats to ecosystems and human health, highlighting the need for innovative approaches to effective remediation, particularly for non-biodegradable emerging pollutants. This research work explores the influence of shape-controlled nanocrystalline titanium dioxide (TiO_2_ NC), synthesized by a simple hydrothermal method, on the photodegradation efficiency of three different classes of emerging environmental pollutants: phenol, pesticides (methomyl), and drugs (sodium diclofenac). Experiments were conducted to assess the influence of the water matrix on treatment efficiency by using ultrapure water and stormwater (basic) collected from an urban drainage system as matrices. The size and shape of the nano-cuboids were accurately controlled during synthesis to assess their impact on photoactivity and selectivity. Regarding total organic carbon removal using TiO_2_ nano-cuboids in basic environments, the results were particularly remarkable. TiO_2_ nano-cuboids and truncated bipyramids synthesized in the 200–250 °C temperature range showed an enhanced photocatalytic efficiency when compared to alternative formulations. Diclofenac, methomyl, and phenol were fully mineralized from ultrapure water and basic stormwater. The TiO_2_ nano-cuboids/nano-bipyramids demonstrated better selectivity and photoactivity in comparison to irregular TiO_2_ nanoparticles. The differences in photoactivity and selectivity are explained in terms of charge carrier separation and trapping on the different crystal facets. Their performance demonstrates their potential as sustainable materials for the photodegradation of emerging pollutants in various water matrices.

## 1. Introduction

Human activities have resulted in the widespread presence of organic contaminants, such as pesticides, dyes, and drug residues, in soils and waters. New water contaminants classified as emerging concerns are difficult to remove using conventional biological treatment technologies and can be hazardous even at low concentrations [1,2,3]. Therefore, new and more effective tertiary and quaternary water treatment strategies that guarantee the removal and mineralization of these non-biodegradable and polar contaminants, preserving aquatic ecosystems as well as a safe drinking water supply, are urgently needed.

Photocatalysis has emerged as a viable method to treat emerging organic pollutants in recent years. This advanced water treatment technology leverages the power of light-activated catalysts to break down complex and persistent contaminants, offering several advantages, namely low cost, mild operational conditions, no need for chemicals other than oxygen, the possibility to use solar light to activate the catalysts, and the absence of byproducts [4,5,6,7]. Its main drawbacks are a low photonic efficiency caused by fast charge carrier recombination rates and, in liquid systems, the low mass transfer rates [8,9].

The photocatalytic treatment of various contaminants has already been reported in studies considering the effect of different water matrices and the shape of the photocatalyst nanoparticles on the efficiency of several pollutant classes [10,11,12,13,14,15]. It was established that the exposed facets of TiO_2_ have significantly influenced photocatalytic activity and the spatial separation of charge carriers. Different crystal faces can be exposed by controlling the size and shape of photocatalytic materials at the nanoscale, each with distinct surface states and catalytic properties. This nano-structuring can enhance the photocatalytic efficiency of TiO_2_ by improving charge carrier separation and transport across the liquid–solid interface. Several controlled synthesis methods have been attempted in the last several decades to develop TiO_2_ nanostructures with various morphologies, such as sol-gel, hydrothermal, microwave-assisted, and chemical vapor deposition [16,17]. In particular, the cost-effective hydrothermal process produces nanomaterials with a large surface area and a high degree of crystallinity, in contrast to other methods. In particular, TiO_2_ can be successfully produced in a range of morphologies using the hydrothermal approach, including nanowires, nanotubes, nanobelts, nanorods, nanosheets, nanoflowers, and hierarchical microspheres [17,18,19,20,21,22]. The control of the shape and size of TiO_2_ nanoparticles allows the study of structure/photoactivity relationships, unravelling the understanding of various insights affecting the process (e.g., photogenerated charge carrier separation and trapping, and the role of the adsorption of ions and substrates) and enabling the tuning and optimization of the photocatalytic activity of the material, and, possibly, its selectivity [23,24,25,26,27]. It is worth noting that the shape and exposed facets of TiO_2_ crystals play a crucial role in determining their photoelectrochemical and photocatalytic performance due to differences in surface energy, bandgap alignment, and electronic band topologies [28]. The exposed high-energy facets of TiO_2_ crystals can enhance photocatalytic activity by promoting surface adsorption and interfacial electron transfer. In this regard, nanomaterials with controlled distribution of facets have been recognized as very active in photocatalytic applications spurring the development of new nanomaterials optimized for photocatalytic conversion of new pollutants in diverse water matrices [28].

In this study, we developed a cost-effective hydrothermal synthesis method to produce TiO_2_ nano-cuboids with varied morphologies and high crystallinity. The one-pot hydrothermal approach utilized titanium tetraisopropoxide (TTIP) as the titanium precursor and acetic acid as a capping agent to control the shape of the nanoparticles. Hydrothermal conditions, such as temperature and treatment time, were optimized to tailor the surface morphologies and achieve distinct structural features. To evaluate the photocatalytic efficiency, the shape-controlled catalysts were evaluated in different water matrices (ultrapure and basic stormwater). The diclofenac, methomyl, and phenol were selected as model pollutants in this study due to their prevalence in water systems, diverse chemical structures, and hazardous impacts (Table S1). Diclofenac, a widely used anti-inflammatory drug, is increasingly detected in freshwater systems, where it poses toxicity risks to aquatic organisms. Its aromatic structure and functional groups, as well as the presence of chlorine, contribute to its resistance to conventional treatment methods, often resulting in removal efficiencies ranging from 30% to 70% [29,30]. Methomyl, a carbamate-based insecticide, is highly soluble in water, leading to contamination of both ground and surface waters. The thiol ester group in its structure plays a key role in its chemical stability and degradation pathways, while its low sorption affinity to soils ensures its high mobility in aquifers, raising concerns about its long-term environmental effects [31,32]. Phenol and its derivatives are well known for their bio-recalcitrant and acute toxicity and are commonly found in industrial wastewater [33]. These pollutants were chosen to represent a broad spectrum of chemical complexities and environmental concerns, providing a diverse set of candidates to evaluate the performance of the synthesized photocatalyst. TiO_2_ nano-cuboids with varying morphologies were characterized using X-ray diffraction (XRD), field emission scanning electron microscopy (FESEM), X-ray photoelectron spectroscopy (XPS), Fourier transform infrared spectroscopy (FTIR) and UV–visible diffuse reflectance spectroscopy (UV-Vis-DRS), and their photocatalytic performance was evaluated by degrading phenol, methomyl, and diclofenac in stormwater and Milli-Q water under UV light. The correlation between controlled morphology and pollutant degradation provides valuable insights for designing next-generation photocatalysts, highlighting the noteworthy potential of nano-cuboid/nano-bipyramid-based systems for efficient pollutant removal.

## 2. Results and Discussion

### 2.1. Structural Analysis

**Figure 1:**
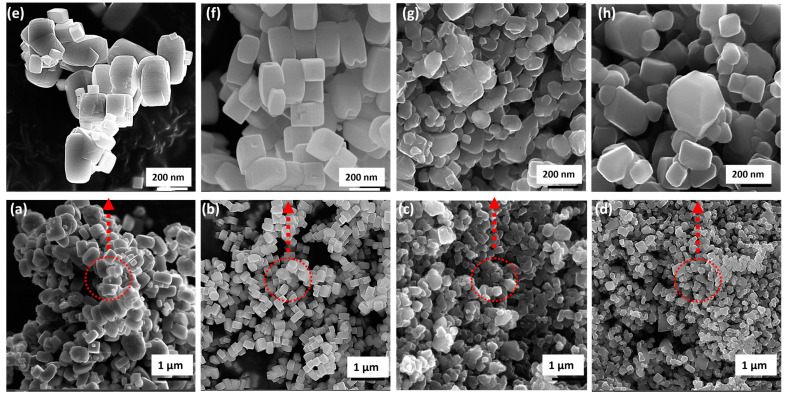
FESEM images of TiO_2_ nano-cuboids prepared under different reaction temperature conditions at 24 h: (**a**,**e**) TN-175 °C; (**b**,**f**) TN-200 °C; (**c**,**g**) TN-225 °C; and (**d**,**h**) TN-250 °C.

Figure 1 shows field emission scanning electron microscopy (FESEM) images of synthesized TiO_2_ nanoparticles, where the reaction temperature exhibited a considerable impact on the shape of the particles and the developed TiO_2_ nanostructures transformed from nano-cuboid-shaped particles to truncated bipyramids. Specifically, the particles transformed from larger nano-cuboids at a temperature of 175 °C into well-defined cuboidal shapes with good size uniformity at 200 °C (TN-200 °C). This suggests that the reaction temperature plays a critical role in determining the shape of the TiO_2_ particles and that a 200 °C temperature leads to a more uniform and defined cuboidal shape. When TiO_2_ is hydrothermally synthesized at 225 °C (TN-250 °C), the crystallite shapes start to distort, and at 250 °C, they transform into truncated bipyramids. This suggests that the TiO_2_ crystals are undergoing a morphological transition, resulting in a change in their shape and structure. It is worth noting that such changes in the morphology of TiO_2_ can significantly affect its properties and, therefore, can be considered a potential photocatalyst (discussed in a later section). The high-resolution FESEM images highlight evidence that the tetragonal nano-cuboids have characteristic square-like top facets and bar-like lateral facets. A [001]-elongated anatase single crystal with four {100} lateral facets and two {001} top facets can be seen if we concentrate on the image of a single nano-cuboid lying on its one lateral facet, which shows sharp diffraction spots attributed to the [010] zone axis of the tetragonal anatase TiO_2_ crystal. The two flat square surfaces are designated as the {001} facets, while the remaining four rectangular surfaces are referred to as the {100} facets, based on the symmetry of the well-defined anatase TiO_2_ crystals.

**Figure 2:**
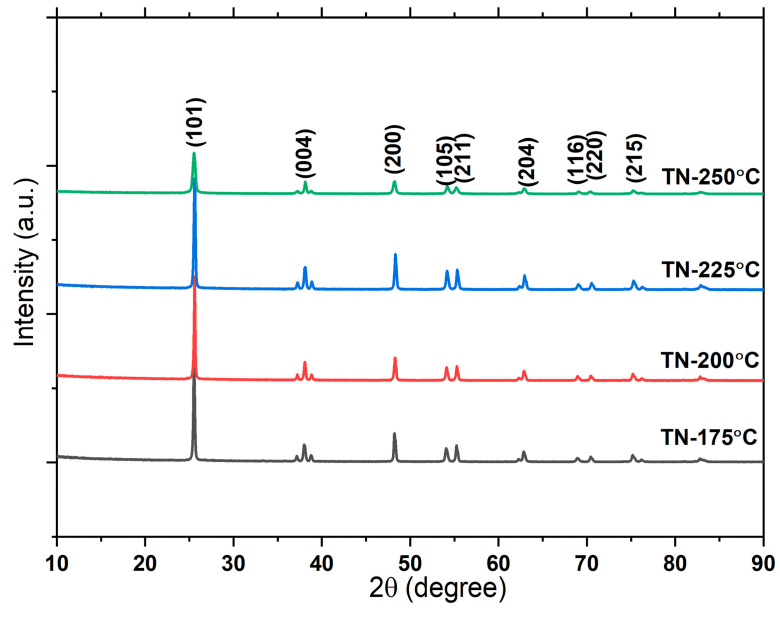
XRD patterns of TiO2 nano-cuboids prepared under different conditions.

The X-ray diffraction (XRD) patterns of the TiO_2_ NCs developed under various temperature conditions are reported in Figure 2. The nano-cuboid TiO_2_ displays characteristic diffraction peaks at 2θ values of 25.3°, 37.8°, 48.0°, 53.9°, 55.0°, 62.7°, 68.7°, 70.3°, and 75.0°, corresponding to the (101), (004), (200), (105), (211), (204), (116), (220), and (215) planes of tetragonal anatase TiO_2_ (JCPDS card no. 21-1272). Comparatively, the TiO_2_ NCs developed under certain reaction temperatures exhibited comparable diffraction patterns, with slight variations in peak intensity for all planes. With the increase in reaction temperature, the (101) peak intensity varies, where the TN-250 °C specimen shows a relatively less intense broader peak intensity, possibly due to the presence of mixed morphologies such as nano-cuboids and truncated pyramids. Furthermore, for comparatively small nanocrystals, diffraction peaks can broaden and shift due to slight changes in the interatomic distances (TN-225 °C). However, no other significant changes are observed.

**Figure 3:**
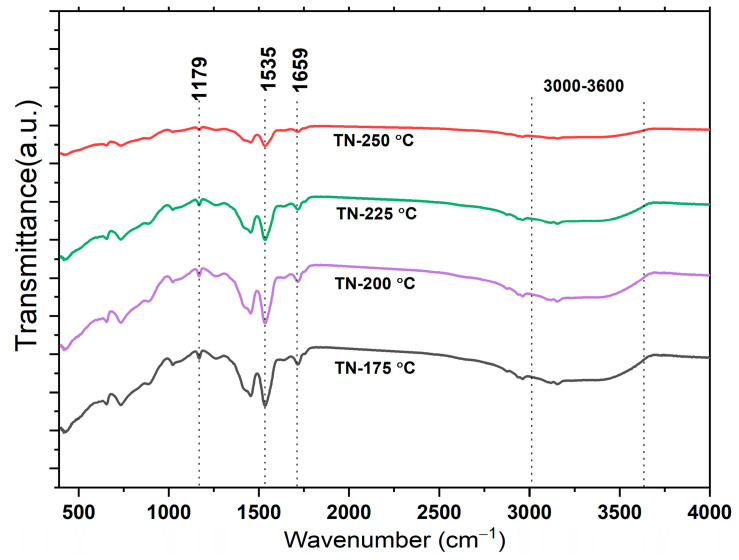
FTIR spectra of developed nano-cuboid TiO_2_ under different reaction conditions.

Figure 3 displays the Fourier transform infrared spectroscopy (FTIR) spectra of TiO_2_ NCs synthesized at various temperatures for 24 h. Ti-O stretching and Ti-O-Ti bridging stretching modes are responsible for the bands seen between 400 and 1000 cm^−1^. The stretching of the O-H mode of the hydroxyl group causes the broadband between 3000 and 3600 cm^−1^. The band at 1659 cm^−1^ for the TiO_2_ nanoparticles is attributable to the O C-O-Ti link of the carbonate structure [28]. The 1535 cm^−1^ band represents N-H in-plane bending and C-N stretching vibrations, while the 1179 cm^−1^ band is caused by C-N stretching [34]. There are no nitrogen-related bands in TiO_2_, implying that the N species are interstitial. As the reaction temperature rises from 175 °C to 250 °C, the O-H stretching mode of the hydroxyl group becomes weaker.

**Figure 4:**
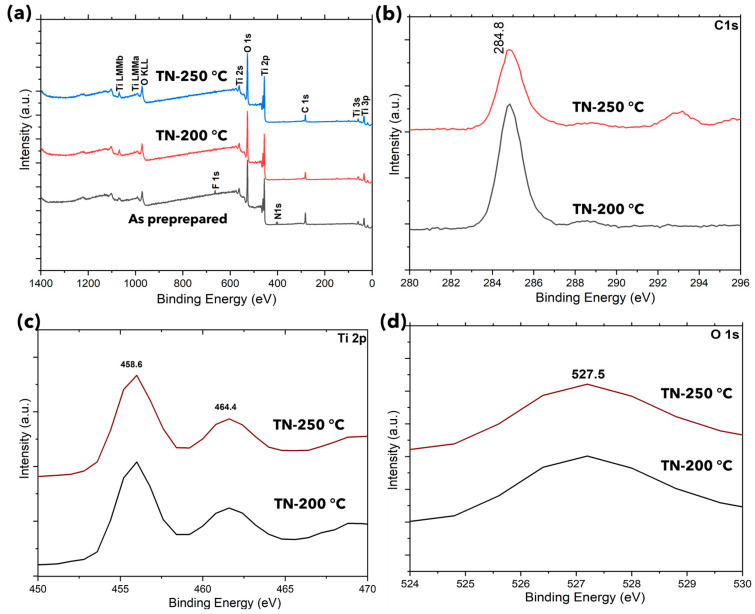
XPS spectra of TiO_2_ nano-cuboids and nano-bipyramids: (**a**) full scan, (**b**) C 1s, **(c)** Ti 2p, (**d**) O 1s.

For better comparison, X-ray photoelectron spectroscopy (XPS) analysis is conducted on the specimens developed at 200 °C and 250 °C and the specimen without post-treatment (see Section 3.2) to understand the role of the selected synthesis solution (TN-200 °C, TN-250 °C) on their structural compositions (Figure 4). The broad scan survey spectrum indicated that TiO_2_ particles include C, N, Ti, and O elements, with photoelectron peaks with binding energies of 284.8 (C 1s), 400.09 (N 1s), 458.8 (Ti 2p), and 527.5 (O 1s), respectively. The untreated material showed the 683.88 eV (F 1s) peak, demonstrating the removal of surface fluorine and other residual organic compounds following post-treatment. Ionic liquids are an environmentally friendly and operationally safe alternative to other corrosive fluoride sources, such as HF [35]. The [bmim]^+^ ion adsorbed on the surface of TiO_2_ nanocrystals is responsible for the N 1s peak at 401.1 eV. The nitrogen atoms in the C-N bonds may come from the capping agent or the precursor. Moreover, the C–O bond is responsible for the weak peak at 284.8 eV (Figure 4b) [36,37]. Numerous studies have connected carbonate species, namely Ti-O-C, on the TiO_2_ surface to C dopants. On the other hand, substitutional C-doping can be excluded, due to the absence of a peak at 281–282 eV, which is characteristic of Ti-C bonding (O-Ti-C linkage). According to Figure 4c, the binding energies of Ti 2p_3/2_ and Ti 2p_1/2_ are 458.8 and 464.4 eV, respectively. This suggests that the surface Ti oxidation state is identical to that of bulk TiO_2_ [37,38,39]. As seen by the approximately 5.7 eV split between the Ti 2p_1/2_ and Ti 2p_3/2_ core levels, the Ti^4+^ in the TiO_2_ is in a normal state. It is important to note that although the different calcination temperatures lead to the significant surface morphological refinement of TiO_2_ particles, from nano-cuboids to nano-bipyramids, their chemical composition is comparable for both types of surface morphologies.

### 2.2. UV–Visible DRS Spectra

**Figure 5:**
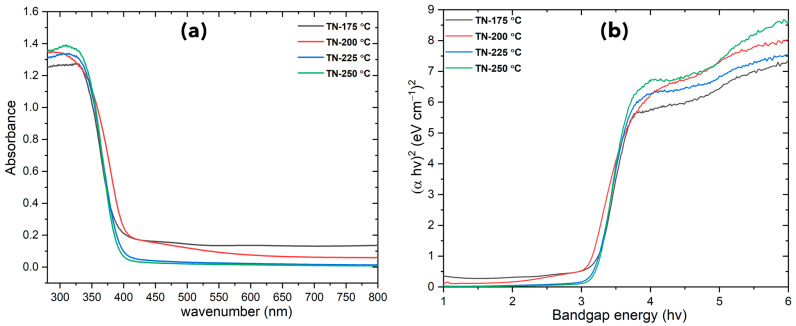
(**a**) UV-Vis absorption spectra, (**b**) bandgap of developed TiO_2_ nano-cuboids.

The diffuse reflectance spectrum of TiO_2_ nano-cuboids is shown in Figure 5a, with the fundamental absorption edge located at 385–400 nm. For the TN-200 °C, there is a noticeable shift in the absorption edge into the visible light area (wavelengths longer than 400 nm). This shift could be explained by the existence of oxygen vacancy states, which result from the production of Ti^3+^ species inside the TiO_2_ lattice.

The bandgap energies (E_g_) of synthesized TiO_2_ NCs may be determined using Tauc plots, taking into account that TiO_2_ is an indirect bandgap material. Figure 5b depicts a plot of (αhv)^2^ against hν, where α is the absorption coefficient and hν is the photon energy. The bandgaps of the TiO_2_ NCs synthesized at 175 °C, 200 °C, 225 °C, and 250 °C were 3.16, 3.05, 3.14, and 3.12 eV, respectively. It was found that surface carbonaceous and carbonate species, as well as interstitial nitrogen species (Ti-O-N), did not significantly alter the TiO_2_ bandgap. What should be underlined is that, in comparison to structural studies (XRD), the analysis of the pure anatase form demonstrates a smaller bandgap, which can lead to absorption in the visible energy range, thereby increasing TiO_2_ photoconversion efficiency.

### 2.3. Photoactivity of Anatase Nano-Cuboids and Nano-Bipyramids

The photoactivity assessments of the different samples were carried out measuring the photoconversion rates of three distinct pollutants under UV irradiation (385 nm). The measurements were performed over an irradiation time of 120 min, and TOC measurements were conducted before and after the end of the degradation experiment. Preliminary adsorption tests were carried out in dark conditions for 30 min; these experiments showed a negligible amount of adsorption on these catalysts (<1%). The reaction kinetics in the photodegradation (first-order kinetics) was also calculated (Tables S2–S4) by the Langmuir–Hinshelwood model (low coverage case), fitting the time profile data with Equation (1).

(1) $$-\ln\left(\frac{C}{C_{0}}\right)=k_{r}k_{ad}t$$

where *k_r_* = intrinsic rate constant, *k_ad_* = adsorption equilibrium constant, *t* = time, and *C*_0_ represents the initial pollutant concentration in the system. The obtained starting rate values were utilized to compare the photocatalytic process’s efficiency across varied reaction circumstances.

#### 2.3.1. Photodegradation of Phenol

**Figure 6:**
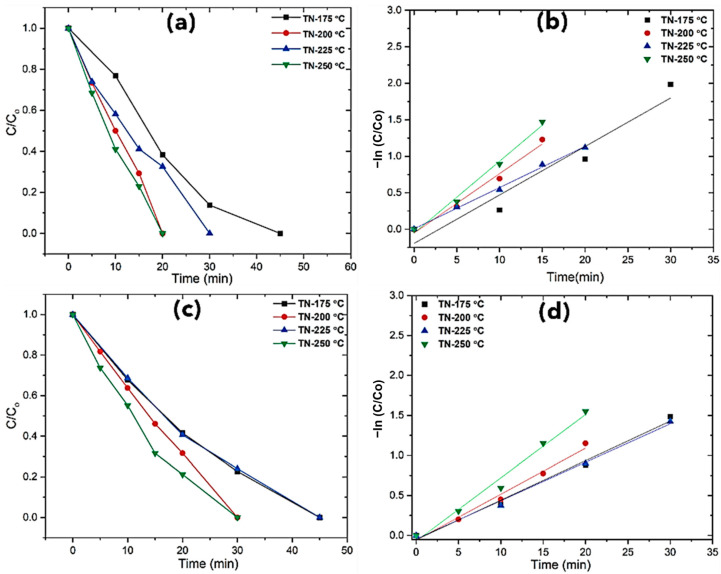
Phenol photodegradation of developed TiO_2_ and pseudo-first-order kinetics plots: (**a**,**b**) ultrapure water (UW), (**c**,**d**) harvested stormwater (SW).

TiO_2_ photocatalysis is able to convert phenol into carbon dioxide and water. The primary photoconversion intermediates are di-hydroxylated derivatives and quinones (e.g., catechol and benzoquinone) and low amounts of condensation products (e.g., phenoxyphenols). Orto dihydroxy derivatives then undergo ring opening with the formation of dicarboxylic acids. In the mineralization phase, these compounds are mineralized to CO_2_ and H_2_O [40], as confirmed by total organic carbon (TOC) measurements. To explore its effectiveness, reactions were carried out with developed morphologies of nano-cuboids, in two different water matrices (ultrapure water (UW) and highly basic stormwater (SW)) and at the 0.1 mM fixed concentrations of phenol. The catalytic performance of the cuboids and truncated bipyramids obtained at 200 °C and 250 °C, respectively, was much better with respect to the corresponding nanomaterials synthesized at a lower temperature (Figure 6). Both catalysts showed the best photodegradation efficiency within 20 min (UW), as shown in Figure 6a, and complete degradation was achieved in 30 min in the case of rainwater, which might have been due to the presence of salts; however, it was still significant. All the selected catalysts demonstrated substantial degradation of phenol in just 20–45 min in both water matrices. The higher surface area of the nanoparticles and active sites is what causes the efficient degrading behavior. Better degradation could be due to the larger levels of reactive hydroxyl radicals caused by an increase in electron–hole pairs on the catalyst surface. A slower rate of photogenerated charge recombination, improved light absorption by the materials, and improved interfacial charge transfer are some of the possible causes of this increased activity [41,42]. First-order kinetics concerning phenol concentrations were found to fit all the experimental data, and first-order rate constants were estimated. The rate of degradation of phenol was also calculated by the Langmuir–Hinshelwood model. Table S2 (Supplementary data) illustrates the results obtained for the photocatalytic treatment of the target pollutant in UW as well as in SW.

#### 2.3.2. Photodegradation of Methomyl

**Figure 7:**
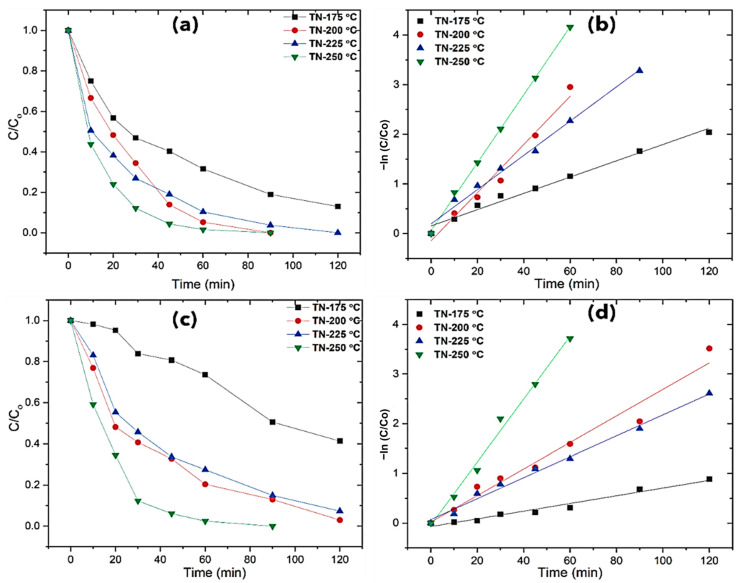
Methomyl photodegradation of developed TiO_2_ and pseudo-first-order kinetics plots: (**a**,**b**) ultrapure water (UW), (**c**,**d**) stormwater (SW).

The influence of the water matrix on pesticide (methomyl) photodegradation in the presence of TiO_2_ nano-cuboids under UV irradiation is shown in Figure 7. Some important differences can be observed. Almost all the pesticide was completely degraded in ultrapure water after 90 min of irradiation for TN-200 °C and for TN-250 °C (Figure 7). Conversely, comparatively slower pesticide degradation was always addressed (120 min) when rainwater was used. In terms of mineralization, sufficient TOC conversions were obtained for TN-200 °C and TN-250 °C, with values of 41% and 55% for ultrapure water and 25% and 39% for rainwater after 120 min, respectively; this was most likely due to the presence of refractory byproducts in aqueous solution. The corresponding degradation curves were fitted to pseudo-first-order kinetic constants, assuming that a modified Langmuir–Hinshelwood kinetic model adequately described the kinetics of this photocatalytic process, allowing the apparent kinetic constant, k_pesticide_, to be calculated for each catalyst. Table S3 (Supplementary data) shows that all the nanoparticles had high coefficients of determination (r^2^ = 0.9726–0.9995). The acquired results were comparably significant when compared to comparable findings in previous literature investigations [43,44,45].

#### 2.3.3. Photodegradation of Sodium Diclofenac

**Figure 8:**
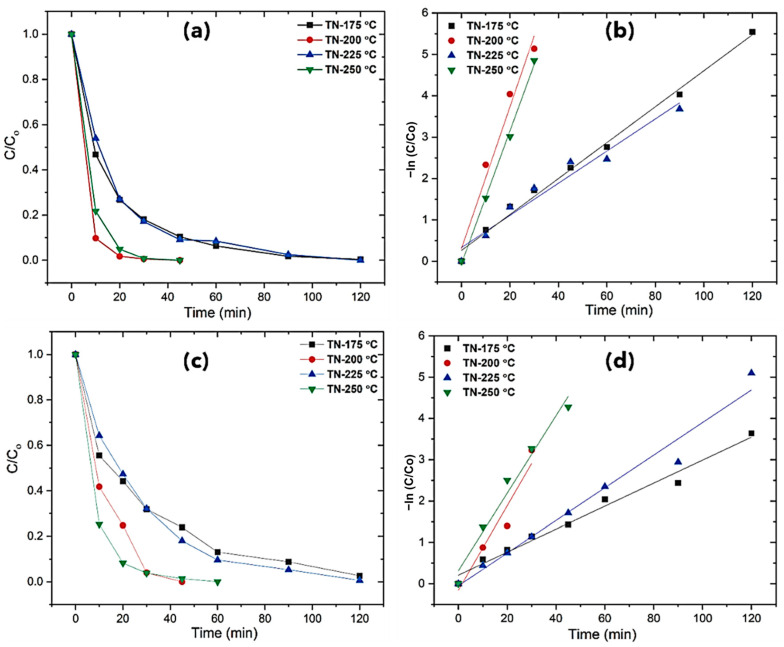
Diclofenac photodegradation of developed TiO_2_ and pseudo-first-order kinetics plots: (**a**,**b**) ultrapure water (UW), (**c**,**d**) harvested stormwater (SW).

As was previously discussed, diclofenac breaks down under photocatalytic treatment into a variety of species, resulting in the identification of many hydroxy- and dihydroxy-diclofenac derivatives, which were then further converted into derivatives of chloro- or hydroxyl-phenol. Complete mineralization is eventually attained through the ring opening, which leads to the production of dicarboxylic acids [46,47,48]. Figure 8 depicts the time profiles of the DCF concentration in ultrapure water and rainfall exposed to UV radiation. During the experiment, it was observed that the degradation of diclofenac was minimal in the absence of a photocatalyst. The degradation rates measured in ultrapure water were higher than those obtained with stormwater, which might be attributable to the presence of salts that can adsorb the degradation process. As shown in Figure 8, and as summarized in Table S5, TN-175 °C and TN-225 °C had approximately 99% degradation in 120 min, whereas TN-200 °C and TN-250 °C had remarkably fast degradation in just 45 min in UW, while it was 45 and 60 min in rainwater, respectively. The maximum TOC removal of 79% was achieved with catalyst TN-250 °C in UW, and it was 67% for rainwater, as shown in Table S4 (Supplementary data).

#### 2.3.4. Comparative Analysis of the Photodegradation Efficiency

TiO_2_ has potential as a photocatalyst for degrading organic pollutants; however, it faces challenges due to limited solar light absorption, low quantum efficiency, and inadequate mass transfer in liquid environments. These limitations stem from undesirable properties, fast recombination rates, and colloidal instability of nanosized particles, leading to aggregation and reduced light absorption. However, shape-controlled anatase nano-cuboids (main planes exposed (001) and (100)) and nano-bipyramids (plane exposed (101) and (001)) possibly enhanced charge carrier separation, suppressing electron–hole recombination, and showed relatively good photodegradation efficiency and different selectivity for three different types of pollutants. The nano-cuboids were synthesized via a cost-effective, scalable hydrothermal method, ensuring feasibility for large-scale applications. A surfactant-free synthesis approach minimized harmful additives, emphasizing eco-friendliness. Their compact size, compared to commercial TiO_2_ (Degussa P25), facilitates efficient regeneration and reuse, overcoming challenges like particle agglomeration and recovery issues associated with P25’s small size (10–50 nm). Thermal treatment demonstrated a morphology transition, combining features of nano-cuboids and bipyramidal structures, which formed heterostructures that enhance charge separation and improve photocatalytic activity. These materials provide a sustainable, efficient alternative to P25 for large-scale water treatment technologies.

**Figure 9:**
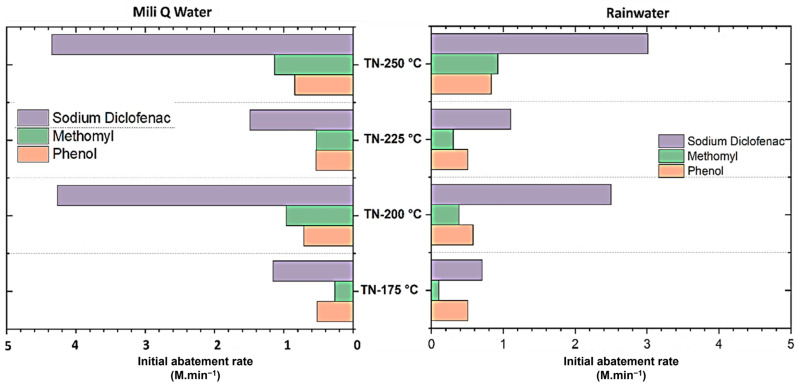
Photodegradation efficiency of pollutants in the presence of anatase nano-cuboids (TN-200 °C) and nano-bipyramids (TN-250 °C) and no shape-controlled nanoparticles (TN-175 °C and 225 °C) under UV irradiation in ultrapure water (UW) and stormwater (SW).

In the treatment of organic pollutants through photocatalysis, the catalyst’s performance varied significantly depending on whether UW or SW effluents were used to prepare the pollutant mixture. The use of UW water generally results in higher photo-efficiency, with a higher TOC removal rate (approximately 79%) and complete degradation of pollutants in just 20–60 min of UV irradiation. The results showed the faster photodegradation of phenol and sodium diclofenac, while the degradation kinetics of methomyl were slightly slower. On the other hand, when the SW matrix was used in the experiment, the TOC removal of all the model pollutants was relatively low, but the photoconversions were still significant compared to the literature studies and thus demonstrated the significance of shape-controlled TiO_2_ nanomaterials (Figure 9).

Two critical factors that greatly influence a catalyst’s photocatalytic efficiency are its specific surface area and crystallinity. More active sites are available on a surface with a larger specific surface area, which allows electrons and holes to go further without recombining. TN-200 °C and TN-250 °C have higher photocatalytic performance than TN-175 °C and TN-225 °C because of their lower dispersity in shape and their improved crystallinity. Improved crystallinity facilitates the charge carriers’ migration to the surface free from defects. These act as recombination sites for carriers produced by photolysis and boost photocatalytic activity. Under UV light, titania absorbs photons (Equation (2)) and ionizes oxygen (Equation (3)). The photo-holes neutralized the (*OH*) groups, producing the hydroxyl radical (*OH^•^*), as shown in the equation (Equation (4)). The resulting *OH^•^* radials can oxidize organic contaminants (Equation (5)) or react directly with holes (Equation (6)) [16,20,21,49]. Nanostructured materials have a high surface area-to-volume ratio and unique properties that enhance photocatalytic treatment and catalytic efficacy.

(2) $$TiO_{2}+hv\to \;e_{CB}^{-}+h_{vB}^{+}\;$$

(3) $$O_{2}+e_{CB}^{-}\;\to +O_{2}^{•-}$$

(4) $${\left(H_{2}O\right)}_{ads}+h_{vb}^{+}\;\to \;H^{+}+OH^{•}$$

(5) $$RH+OH^{•}\;\to \;R^{•}+H_{2}O\;$$

(6) $$RH+h^{+}\;\to \;R^{•}+H^{+}$$

Previous studies have shown that fine-tuning the particle morphology, specifically the exposed specific crystal faces, can enhance the photoactivity of TiO_2_ nanocrystals [50,51]. The reactivity of different faces is related to the type, amount, and location of electronic defects, e^−^ and h^+^. These initial findings suggest promising potential for the obtained anatase TiO_2_ nano-cuboids as photocatalysts. However, a comprehensive examination of the photo-reactivity of these nano-cuboids, considering systematically varied aspect ratios and sizes, is essential to thoroughly assess the facet reactivity order for various photocatalytic reactions. Phenol, with its simple structure and high reactivity, shows the fastest degradation rate (30 min) and nearly complete mineralization under UV light with TiO_2_ nano-cuboids, resulting in highly efficient water purification in both Milli-Q and rainwater systems. Diclofenac, an anti-inflammatory drug, degrades at a moderate but promising rate (45 min), achieving effective mineralization while potentially minimizing harmful byproducts, such as hydroxy-diclofenac and dichlorodiclofenac [52], as confirmed by TOC analysis. Methomyl, a pesticide, follows a more complex degradation pathway [53] and achieves complete mineralization within 90 min under similar conditions. TiO_2_ nano-cuboids demonstrate excellent photocatalytic performance across various pollutants, with photodegradation occurring within 30–90 min in both Milli-Q and rainwater. Although a comprehensive evaluation of TiO_2_ nano-cuboids versus P25 requires that factors such as byproduct formation, irradiation intensity, regeneration, and reaction conditions are accounted for, both materials effectively degrade phenol. P25 (C_0_ = 0.53 mM, 0.5 g/L TiO_2_) achieves full degradation in 180 min [54], whereas nano-cuboids (C_0_ = 0.1 mM, 1 g/L TiO_2_) achieve this in 30 min. The nano-cuboids show superior total organic carbon (TOC) removal [55], indicating more complete mineralization. Their unique shape-controlled morphology (size > 40 nm) increases light harvesting, improves charge separation, and enhances electron transfer, significantly reducing electron–hole recombination. These properties make TiO_2_ nano-cuboids potentially sustainable alternatives for high-performance environmental remediation. However, further studies are required to evaluate additional parameters, such as byproduct formation, regeneration efficiency, and long-term stability, to fully assess the potential of TiO_2_ nano-cuboids as a viable alternative to commercial TiO_2_ for environmental remediation applications.

## 3. Materials and Methods

### 3.1. Materials

Alfa Aesar (Ward Hill, Massachusetts, U.S) provided the butyl-3-methylimidazolium tetrafluoroborate ([bmim][BF4]) ionic liquid. Acros provided titanium tetraisopropoxide (TTIP), while Beijing Chemical Works (Gongye Dongqu, Anding Town, Daxing Dist., Beijng, China) supplied acetic acid (HAc) and hydrofluoric acid (40%). Scharlab (Barcelona, Spain) provided methanol and orthophosphoric acid of HPLC quality for conducting chromatographic investigations. Every solution was made using Milli-Q Type 1 water. The pesticide used for treatment was Aragonesas Agro S.A. (Madrid, Spain) 99.5% pure methomyl. The selected drug was sodium diclofenac (DCF, >99% purity), and phenol (99% purity) were supplied by Aldrich (Darmstadt, Germany). Table S1 (Supplementary data) lists the investigated pollutants’ general characteristics.

### 3.2. Synthesis of Titanium Dioxide Nano-Cuboids

Anatase TiO_2_ nano-cuboids/nano-bipyramids of varying sizes were developed using a quaternary solution system made up of TTIP, deionized water, [bmim][BF_4_], and HAc. To synthesize, 40 mL of Hac, 100 μL of water, and 1.2 mL of [bmim][BF_4_] were combined in a 100 mL Teflon autoclave and then mixed with 1 mL of TTIP. Reaction time and temperature were shown to have a substantial influence on the controlled shape structure when the mixture was held for different times (temperatures) for microstructure refinement. The reaction time and temperature were initially methodically adjusted during the synthesis to achieve anatase TiO_2_ nano-cuboids of distinct sizes and shapes (controlled facets). This work concludes the four unique shapes, and their photoactivity, obtained at reaction temperatures of 175 °C, 200 °C, 225 °C, and 250 °C over a constant period of 24 h. The materials are termed TN-175 °C, TN-200 °C, TN-225 °C, and TN-250 °C, respectively. The resultant white powder was separated by centrifugation and then rinsed with ethanol and water before being freeze-dried for a whole night. To produce pure, fluorine-free anatase nano-cuboids, surface fluorine and other leftover organic compounds were removed using a heat treatment procedure that required a time period of three hours at 800 °C. As a reference, the specimen without calcination (800 °C) was also analyzed. Figure S1 (Supplementary data) illustrates the developed nano-cuboids’ synthesis process.

### 3.3. Characterization

The morphology and microstructural characteristics were analyzed using a SEM (JEOL-IT300 microscope coupled with an EDS detector, JEOL Ldt, Tokyo, Japan). The XRD diffractograms were recorded using the X’Pert High Score diffractometer (Rigaku, Tokyo, Japan), utilizing a copper K-α (λ = 1.54 Å−1) emission source at 10 mA and 30 kV. Diffractograms were recorded in the 2θ range of 10–90° with a step size of 0.017°. Fourier transform infrared spectroscopy (FTIR) (Excalibur Series instrument, Agilent Technologies, Santa Clara CA, USA) in the ATR mode was used to analyze the surface functional group and chemical bonding of the samples in the range of 550 to 4000 cm^−1^ with a 4 cm^−1^ resolution and 32 scans using a diamond crystal as an internal reflective element (IRE). Thermo Scientific (Waltam MA, USA) ESCALAB 250Xi X-ray spectrometer was utilized to conduct XPS using Al Kα radiation at 1486.6 eV. A Shimadzu (Kyoto, Japan) UV-Vis-NIR spectrophotometer (Model UV-3600) was used to collect UV-Vis diffuse reflectance spectra from 200 to 800 nm at ambient temperature.

### 3.4. Photocatalytic Efficiency Assessment

The catalyst’s photocatalytic activity was measured by exposing suspensions (with a catalyst loading of 1 g L^−1^) in the photoreactor. The irradiation source used was a 385 nm UVA lamp with 10 LEDs (Seoul Viosys, Seoul, Republic of Korea) scattered to equally cover the reactor surface. Each LED lamp setup used a current intensity of 250 mA, which is equivalent to the consumption of 8.38 W of electrical power by 385 nm LED lights. The lamp was placed 4.5 cm away from the water surface. The actual radiant power under this experimental condition was determined using the potassium ferrioxalate actinometry method. The results showed that the 385 nm LEDs emitted 1682.8 ± 77.1 μmol m^−2^ s^−1^ photons. With a starting pollutant concentration of 0.1 mM, the photocatalytic activity was determined by fitting disappearance curves to an exponential decay. The pH was measured at its normal level. After the reaction mixture was mechanically stirred and left in the dark for half an hour, the pollutant adsorption in the material was assessed. Then, 0.8 mL aliquots of the irradiation solution were taken with a syringe fitted with a nylon filter (0.45 μm pore size) to prevent suspended photocatalyst particles from monitoring the reaction’s progress. The contaminant’s content was evaluated using an Agilent Technologies (Santa Clara, CA, USA) HPLC chromatograph 1200 Series, which included a diode array detector, a binary gradient high-pressure pump, and an automated sampler. Isocratic elution was carried out using a 20/80 acetonitrile/formic acid aqueous solution (0.05% *w*/*v*) at a flow rate of 0.5 mL/min and injection volume of 20 µL. The column used was Kinetex C18 150-2 (150 mm length, 2 mm I.D., 2.6 µm core–shell particles, Phenomenex, Torrance, CA, USA).

For diclofenac, elution was performed using an 80/20 0.1% orthophosphoric acid/acetonitrile aqueous solution. The detection was carried out at 220 nm for phenol and 222 nm for sodium diclofenac. For methomyl, the column used was the Ultrasep ES Pest with dimensions of 250 × 3 mm; the particle size was 5 µm, and the pressure was 400 bar. Elution was performed using 10% acetonitrile/0.1% orthophosphoric acid, and detection was carried out at 231 nm.

Following complete irradiation, the samples were taken out to measure the TOC content in the reaction medium. These samples were then examined using a TOC-VCSH/CSN Shimadzu analyzer. The water sources used for the studies were Milli-Q ultrapure water (pH = 6.5) and stormwater (pH = 8.1–8.6), which was collected from the retention tank of a sustainable urban drainage system (SUDS) established in Madrid, Spain. Table S5 of the Supplementary data provides an analysis of the physicochemical characteristics of these water samples. Stormwater has a more basic pH range of 8.1–8.6, greater conductivity, and total dissolved solids due to its much higher ion concentration. Rainwater is a heterogeneous electrolyte that contains nitrogen and other nitrogenous compounds, as well as ammonia, nitrate, nitrite, magnesium, calcium, sodium, potassium, chloride, bicarbonate, and sulphate. The catalyst optimization was first conducted on phenol, which served as a model pollutant, to determine the optimal catalyst concentration (1 g/L). This concentration was then applied to the degradation of all three pollutants—phenol, methomyl, and diclofenac—each possessing unique and complex molecular structures. This approach allowed a comprehensive comparison, ensuring the effectiveness of the TiO_2_ nano-cuboids across different pollutants. All the studies in this work were conducted in duplicate, and the observed deviations were consistently less than 3%, indicating the high reproducibility and reliability of the results; the single photolysis experiments conducted without any catalyst demonstrated no degradation under the tested conditions, further underscoring the improvement in degradation efficiency achieved with the TiO_2_ nano-cuboids.

## 4. Conclusions

This study highlights the potential of shape-controlled TiO_2_, in which nanoparticles with low shape dispersity (nano-cuboids and nano-bipyramids) showed greater photoactivity due to fewer defects, fewer recombination sites, and higher crystallinity. The photoactivity was about twice as high in the samples with uncontrolled TiO_2_ shapes. Notably, the TN-250 °C and TN-200 °C catalysts exhibited superior performance in photodegradation and total organic carbon (TOC) abatement compared to TN-175 °C and TN-225 °C. Among the shape-controlled TN-250 (truncated bipyramids) and TN-200 (cuboids) nanoparticles, the TN-250 exhibited higher activity. This behavior confirms that the facet couple {101} and {001} leads to improved charge separation with respect to the couple {010} and {001}. Due to the superior photocatalytic properties of anatase, the nano-cuboids/nano-bipyramids offer a distinct advantage, successfully mineralizing three emerging pollutants—phenol, methomyl, and diclofenac—in two different water systems: harvested stormwater and ultrapure water. This study emphasizes the effectiveness of shape-controlled nanoparticles to increase photo-efficiency in the conversion of complex contaminants in water systems, including harvested stormwater for reuse applications. From a technical perspective, optimizing catalyst loading to improve photocatalytic efficiency for individual pollutants, coupled with assessing the catalyst’s reusability, is a crucial step toward the further development of nano-cuboids/nano-bipyramids as efficient solutions for pollutant degradation in various environmental applications.

## Data Availability

Data are contained within the article and Supplementary Materials.

## Supplementary Materials

The following supporting information can be downloaded at: https://www.mdpi.com/article/10.3390/molecules30020424/s1, Figure S1: Schematic route of the preparation of the TiO_2_ nanocuboids (TN-200 °C); Table S1: General characteristics of the pollutants investigated in this work; Table S2: Constant rate (k) and decomposition rate (χ) of phenol in the presence of TiO_2_-based nanocuboids; Table S3: Constant rate (k) and decomposition rate (χ) of Methomyl in the presence of TiO_2_-based nanomaterials; Table S4: Constant rate (k) and decomposition rate (χ) of Diclofenac in the presence of TiO_2_-based nanomaterials; Table S5: Physicochemical parameters of the two assessed water matrices. The supplementary file is included to address the details of experimental additional information and photodegradation data of emerging pollutants.
