# Supplementary material for: From Anatase TiO2 Nano-Cuboids to Nano-Bipyramids: Influence of Particle Shape on the TiO2 Photocatalytic Degradation of Emerging Contaminants in Contrasted Water Matrices
Source: Molecules. 2025 Jan 20;30(2):424. doi: 10.3390/molecules30020424 (PMC11767330; doi:10.3390/molecules30020424)
Supplement: Supplementary file 1 [file molecules-30-00424-s001.zip › molecules-3361803-supplementary.pdf]

From Anatase TiO_2 Nano-Cuboids to Nano-Bipyramids: Influence of Particle Shape on the TiO_2 Photocatalytic Degradation of Emerging Contaminants in Contrasted Water Matrices

Humaira Asghar [1], Daphne Hermosilla [2], Francesco Pellegrino [1], Virginia Muelas-Ramos [2], Christian de los Ríos [2],
Antonio Gascó [2], **Valter Maurino [1,*] and Muhammad Ahsan Iqbal [3]**

[1] Department of Chemistry and UNITO-ITT Joint Lab, University of Torino, Via Giuria 7, 10125 Torino, Italy; humaira.asghar@unito.it (H.A.); francesco.pellegrino@unito.it (F.P.)
[2] G-Aqua Research Group, Departamento de Ingeniería y Gestión Forestal y Ambiental, Escuela Técnica
Superior de Ingeniería de Montes, Forestal y del Medio Natural, Universidad Politécnica de Madrid,
C/José Antonio Novais 10, 28040 Madrid, Spain; daphne.hermosilla@upm.es (D.H.); virginia.muelas@upm.es (V.M.-R.); christian.delosrios.quinones@alumnos.upm.es (C.d.l.R.); antonio.gasco@upm.es (A.G.)
[3] Departamento de Ingeniería Química y de Materiales, Facultad de Ciencias Químicas, Universidad Complutense de Madrid, 28040 Madrid, Spain; miqbal@ucm.es
* Correspondence: valter.maurino@unito.it

Table S1. General characteristics of the pollutants investigated in this work.

**Pollutant Empirical formula**	**Chemical structure**	**Molecular weight** (g·mol[-1])	**Half-life** (days)	**Application**
Phenol, C_6H_5OH<br><br>N-(4,6-dimethylpyrimidin-2-yl) aniline	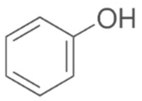	94.11	2-20	- Used in plastic manufacturing industries, phenolic resins, cosmetic industry
Sodium Diclofenac, $C_{14}H_{10}Cl_2NNaO_2$<br><br>2-[(2,6-Dichlorophenyl) amino]benzeneaceticacidsodium salt	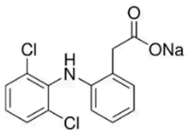	318.13	9-29	- Drug<br>Nonsteroidal anti-inflammatory human and veterinary medicine
Methomyl, $C_5H_{10}N_2O_2S$<br><br>S-methyl (EZ)-N-(methyl carbamoyl oxy) thioacetimidate	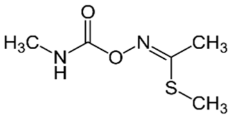	162.2	1.98 - 2.10	- Insecticide<br>Soil-borne insects<br>Sod-turf<br>Leaves (lettuce)<br>Orchards

Table S2. Constant rate (k) and decomposition rate (χ) of phenol in the presence of TiO_2-based nanocuboids.

Sr. No.	Catalyst	Removal Efficiency (%)		Time (min)		k_{Phenol}		r^2		Normalized Degradation Rate ($M.min^{-1}$)		X_{TOC} %	
		UW	SW	UW	SW	UW	SW	UW	SW	UW	SW	UW	SW
1	**TN-175 °C**	100	100	45	45	0.0581	0.0471	0.9596	0.995	0.5204	0.5068	31	25
2	**TN-200 °C**	100	100	20	30	0.0769	0.0535	0.9918	0.9913	0.716	0.5811	57	41
3	**TN-225 °C**	100	100	30	45	0.0568	0.0461	0.999	0.9968	0.5369	0.5053	43	37
4	**TN-250 °C**	100	100	20	30	0.094	0.0744	0.9953	0.9923	0.844	0.8334	69	55

Table S3. Constant rate (k) and decomposition rate (χ) of Methomyl in the presence of TiO_2-based nanomaterials.

Sr. No.	Catalyst	Removal Efficiency (%)		Time (min)		$k_{Methomyl}$		r^2		Normalized Degradation Rate ($M.min^{-1}$)		X_{TOC} %	
		UW	SW	UW	SW	UW	SW	UW	SW	UW	SW	UW	SW
1	TN-175 °C	87	58.69	120	120	0.0164	0.0068	0.9839	0.9726	0.266	0.104	21	13
2	TN-200 °C	100	97	90	120	0.0593	0.0269	0.9913	0.9872	0.968	0.386	41	25
3	TN-225 °C	100	92.65	120	120	0.0345	0.0211	0.9887	0.9922	0.53	0.306	28	17
4	TN-250 °C	100	100	90	90	0.0698	0.0624	0.9995	0.9965	1.136	0.926	55	39

Table S4. Constant rate (k) and decomposition rate (χ) of Diclofenac in the presence of TiO_2-based nanomaterials.

Sr. No.	Catalyst	Removal Efficiency (%)		Time (min)		k_{DCF}		r^2		Normalized Degradation Rate ($M.min^{-1}$)		X_{TOC} %	
		UW	SW	UW	SW	UW	SW	UW	SW	UW	SW	UW	SW
1	**TN-175 °C**	99.6	97.4	120	120	0.0434	0.0278	0.9926	0.9809	1.16	0.7059	25	21
2	**TN-200 °C**	100	100	30	45	0.1843	0.0954	0.9892	0.9692	4.27	2.5	55	42
3	**TN-225 °C**	100	99.38	120	120	0.05645	0.0389	0.9945	0.9888	1.49	1.102	45	35
4	**TN-250 °C**	100	100	45	60	0.1579	0.1093	0.999	0.9846	4.35	3.013	79	67

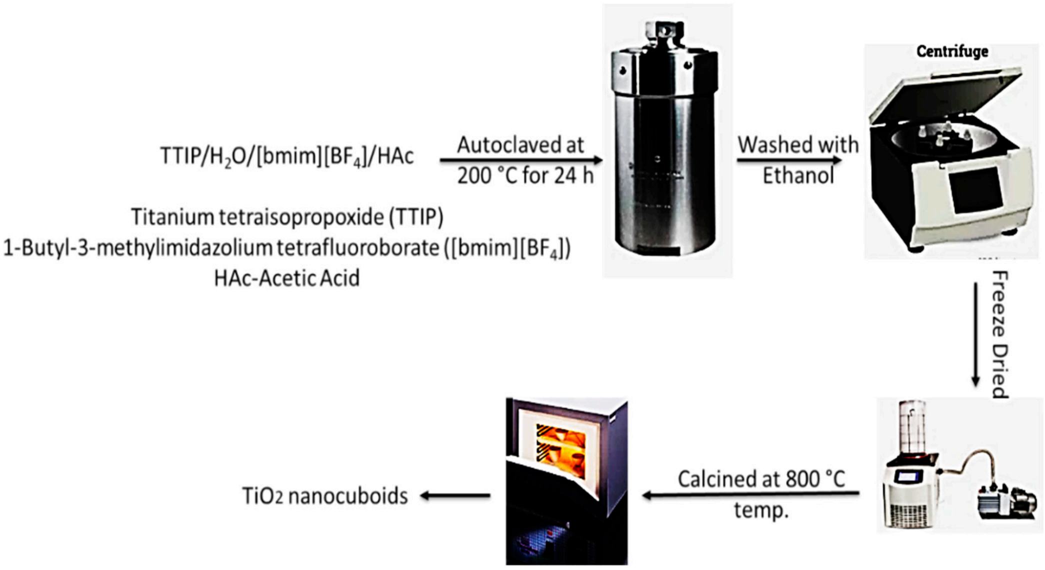

Figure S1. Schematic route of the preparation of the nanocuboids TiO_2 (TN-200 °C).

Table S5. Physicochemical parameters of the two assessed water matrices.

**Parameter**	**Ultrapure Water (UW)**	**Harvested Stormwater (SW)**
pH	6.5	8.1–8.6
**TOC** (mg·L^{-1})	n.d.	< 5.0 ± 0.01
**Conductivity** (mS·cm^{-1})	n.d.	210 ± 2
**CO_3^{2-}** (mg·L^{-1})	n. d.	470 ± 30
**Ca^{2+}** (mg·L^{-1})	n. d.	149 ± 13
**Mg^{2+}** (mg·L^{-1})	n. d.	53.0 ± 3.0
**K^+** (mg·L^{-1})	n. d.	47.0 ± 1.0
**Na^+** (mg·L^{-1})	n. d.	287 ± 56
**Cl^-** (mg·L^{-1})	n. d.	215.6 ± 70
**NO_3^-** (mg N-NO_3^- ·L^{-1})	n. d.	17.0 ± 1.1
**NO_2^-** (mg N-NO_2^- ·L^{-1})	n. d.	< 0.30
**SO_4^{2-}** (mg·L^{-1})	n. d.	37,5 ± 2,4
**PO_4^{3-}** (mg P- PO_4^{3-}·L^{-1})	n. d.	4.3 ± 0.7
**NH_4^+** (mg N- NH_4^+ ·L^{-1})	n. d.	n. d.
**Total dissolved solids** (g·L^{-1})	n. d.	1.00 ± 0.01
